# Butanol production from food waste: a novel process for producing sustainable energy and reducing environmental pollution

**DOI:** 10.1186/s13068-015-0332-x

**Published:** 2015-09-15

**Authors:** Haibo Huang, Vijay Singh, Nasib Qureshi

**Affiliations:** Department of Agricultural and Biological Engineering, University of Illinois at Urbana Champaign, 1304W. Pennsylvania Avenue., Urbana, IL 61801 USA; Bioenergy Research Unit, United States Department of Agriculture, ARS, National Center for Agricultural Utilization Research, 1815N. University Street, Peoria, IL 61604 USA

**Keywords:** Butanol, Food waste, Fermentation, Vacuum stripping, Process integration, Energy

## Abstract

**Background:**

Waste is currently a major problem in the world, both in the developing and the developed countries. Efficient utilization of food waste for fuel and chemical production can positively influence both the energy and environmental sustainability. This study investigated using food waste to produce acetone, butanol, and ethanol (ABE) by *Clostridium beijerinckii* P260.

**Results:**

In control fermentation, 40.5 g/L of glucose (initial glucose 56.7 g/L) was used to produce 14.2 g/L of ABE with a fermentation productivity and a yield of 0.22 g/L/h and 0.35 g/g, respectively. In a similar fermentation 81 g/L of food waste (containing equivalent glucose of 60.1 g/L) was used as substrate, and the culture produced 18.9 g/L ABE with a high ABE productivity of 0.46 g/L/h and a yield of 0.38 g/g. Fermentation of food waste at higher concentrations (129, 181 and 228 g/L) did not remarkably increase ABE production but resulted in high residual glucose due to the culture butanol inhibition. An integrated vacuum stripping system was designed and applied to recover butanol from the fermentation broth simultaneously to relieve the culture butanol inhibition, thereby allowing the fermentation of food waste at high concentrations. ABE fermentation integrated with vacuum stripping successfully recovered the ABE from the fermentation broth and controlled the ABE concentrations below 10 g/L during fermentation when 129 g/L food waste was used. The ABE productivity with vacuum fermentation was 0.49 g/L/h, which was 109 % higher than the control fermentation (glucose based). More importantly, ABE vacuum recovery and fermentation allowed near-complete utilization of the sugars (~98 %) in the broth.

**Conclusions:**

In these studies it was demonstrated that food waste is a superior feedstock for producing butanol using *Clostridium beijerinckii*. Compared to costly glucose, ABE fermentation of food waste has several advantages including lower feedstock cost, higher productivity, and less residual sugars.

## Background

Waste is currently a major problem in the world, both in the developing and the developed countries. Food waste is the single largest component of the waste stream in the United States [1]. According to the US Environmental Protection Agency (EPA), more than 33 million tons of food waste was generated in 2012 alone [2]. The energy embedded in the food waste represented approximately 2 % of annual energy consumption in the United States, which is substantial when compared to other energy conservation and production proposals [3]. Food waste includes unconsumed food that is discarded by food processing industries, retailers, restaurants, and consumers. Despite current large-scale production, many of these food wastes find no current uses different from landfilling or first-generation recycling practices, such as animal feed, composting and incineration [4]. Disposal of food waste in landfill or incineration can cause severe environmental problems, with direct and indirect emissions of greenhouse gases (CH_4_ and CO_2_) [5]. Composting is getting popular, as it diverts food waste from landfill and improves soil structure. However, this type of practice is still carried out at a relatively elevated cost, and has a potential problem of pollution to surface and underground water [6]. In the light of the above comments, effective utilization of food waste for fuels and chemicals will positively influence the energy and environmental sustainability, and the economic competiveness.

Studies have been conducted to process food waste to produce high value-added products (fuels and chemicals), which can be introduced into existing markets [4]. Most of this research has been focusing on anaerobic digestion of food waste to produce biogas [1, 7–9]. Recently, investigations have been conducted to ferment food waste to ethanol, which is mainly used as a transportation biofuel [5, 10–13]. With the increasing interest in biofuel development and the advancement of new biotechnologies, the production of butanol is being developed as a more advanced biofuel to ethanol [14]. Compared to ethanol, butanol has a higher energy content, which makes it a more favorable product as a gasoline blending fuel. Also butanol is better for the existing infrastructure, as it is more hydrophobic, and can be transported via existing pipelines [14, 15]. Since the production of butanol from food grade feedstock (i.e. glucose and corn starch) is expensive, numerous efforts have been made to produce butanol from cellulosic biomass, such as wheat straw [16, 17], corn stover [18, 19], barley straw [20], cassava bagasse [21], switch grass [22], and miscanthus [23]. Liu et al. [24] studied butanol production from wheat bran containing both non-starch polysaccharides and starch. Compared to cellulosic biomass, food waste holds several significant advantages to produce butanol. Firstly, most food waste contains significant amounts of sugars and starch, which can be easily utilized by the butanol-producing culture (*Clostridium*), while cellulosic biomass has to be pretreated under harsh conditions that requires a large amount of energy consumption [25]. Secondly, food waste comprises with significant quantities of functionalized molecules (i.e. proteins, fatty acids, minerals), which can act as nutrients to support the culture growth [4].

The objectives of this study were to investigate the application of food waste as a potential feedstock for butanol fermentation using *Clostridium beijerinckii* P260. Also, an integrated simultaneous saccharification (starch contained in food waste to glucose), fermentation and recovery (SSFR) process was designed and applied to improve the fermentation performance, and to allow the fermentation of food waste at high solids concentrations.

## Results and discussion

### Butanol fermentation in glucose-based medium

**Fig. 1:**
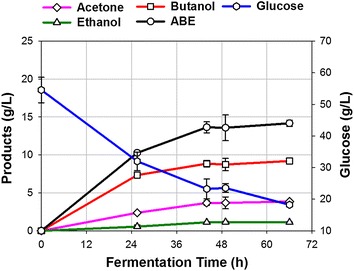
Production of ABE from glucose-based medium in a batch fermentation of *C. beijerinckii* P260

A control ABE (acetone, butanol and ethanol) fermentation experiment with glucose as the substrate was conducted to evaluate performance of *C. beijerinckii* P260 in the batch fermenter. During fermentation, glucose was continuously consumed by the culture, and ABE were continuously produced. Since there was no significant increase in ABE concentration in the broth, the fermentation was intentionally stopped at 66 h (Fig. 1). The culture produced a total of 14.2 g/L ABE during 66 h fermentation time and used 40.5 g/L glucose of the 56.7 g/L present at the beginning of fermentation (Fig. 1). Of the 14.2 g/L produced ABE, the acetone, ethanol and butanol concentrations were 3.9, 1.1, and 9.2 g/L, respectively. At the end of fermentation, the residual glucose concentration in the broth was 16.2 g/L. The major reason for the fermentation cessation before complete utilization of glucose was butanol toxicity to the culture [26]. In this run, the ABE productivity and yield were 0.22 g/L/h and 0.35 g/g, respectively. This productivity and yield was comparable to the previously reported studies [19, 27]. Data collected in the control experiment were employed as the baseline for the evaluations of food waste fermentations.

### Butanol fermentation in food waste medium

**Fig. 2:**
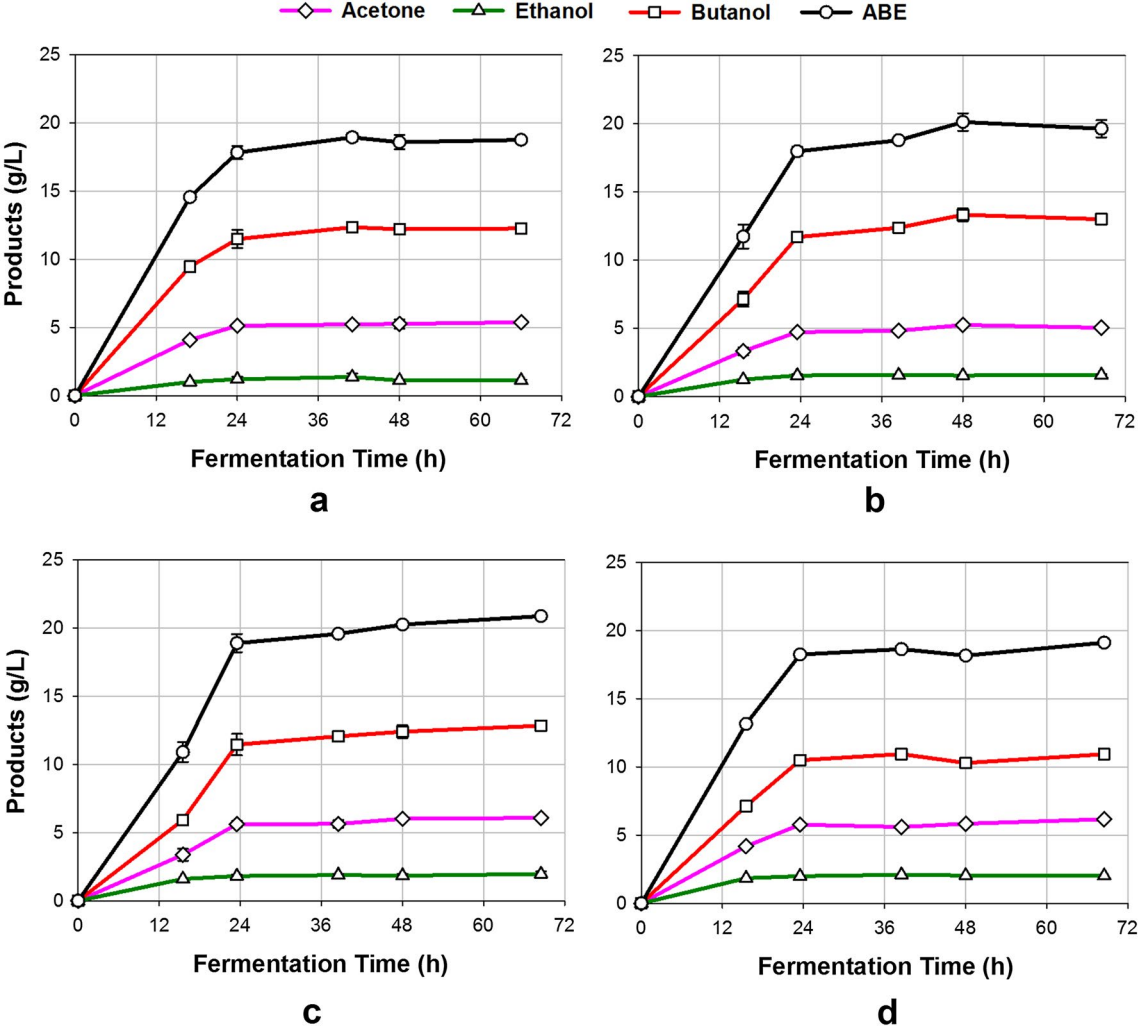
Production of ABE at various initial concentrations of food wastes in medium using *C. beijerinckii* P260. **a** food waste concentration 81 g/L; **b** food waste concentration 129 g/L; **c** food waste concentration 181 g/L; **d** food waste concentration 228 g/L

**Fig. 3:**
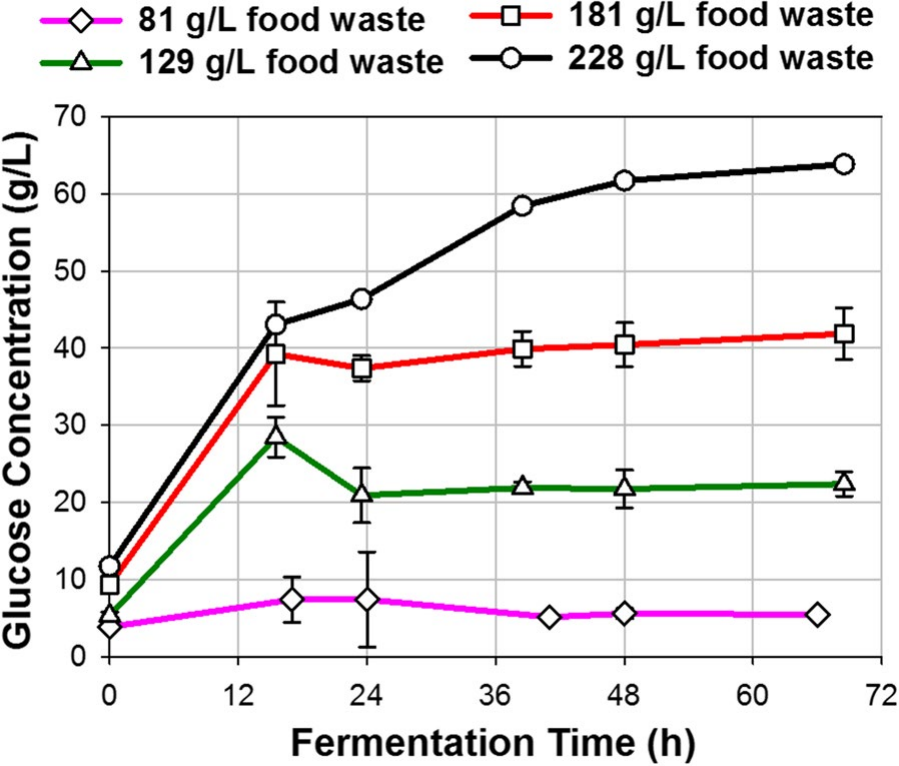
Glucose concentrations in broth during ABE fermentation with various initial concentrations of food wastes

ABE fermentations were performed with food waste medium at different concentrations. For ABE fermentation with food waste at an initial concentration of 81 g/L (containing 60.1 g/L equivalent glucose), the fermentation was very vigorous between 12 and 24 h. At 41 h, the fermentation was complete and no further ABE was produced. At that time, total ABE in the fermentation broth was 18.9 g/L of which acetone, ethanol, and butanol were 5.2, 1.4 and 12.3 g/L, respectively (Fig. 2a). Based on a fermentation time of 41 h (when the culture stopped producing ABE), a productivity of 0.46 g/L/h was obtained (Table 1). This productivity was over 100 % higher than the control experiment, where 56.7 g/L glucose was added at the beginning of fermentation. It is considered that food waste contained some unknown chemicals/compounds that stimulated ABE production. After fermentation, the residual glucose was 5.4 g/L (Fig. 3). The incomplete utilization of glucose was probably due to the product inhibition to the culture, since the butanol concentration was high at 12.3 g/L. The residual starch in the broth was 5.3 g/L (Table 1), indicating that *C. beijerinckii* was unable to hydrolyze the food waste starch completely to glucose. *C. beijerinckii* is known to hydrolyze corn starch to glucose and for this reason no external amylolytic enzymes were added to the fermenters. Based on the above data, it is recommended to add some external enzymes to help hydrolyze the unhydrolyzable residual starch, such as Granular Starch Hydrolyzing Enzyme [28]. By taking considerations of residual glucose and starch content in food waste broth after 41 h fermentation, a total of 49.3 g/L glucose was consumed by the culture. Therefore, the ABE yield was 0.38 g/g, which was comparable to the yield of ABE fermentation with glucose medium in the control experiment. Food waste is expected to have several advantages including lower cost than glucose, higher product concentration in broth, higher productivity, and less residual sugars. This suggests that food waste is a superior feedstock for butanol production.

**Table 1:** A summary of ABE production from glucose and food wastes at various levels using *C. beijerinckii* P260

Substrate	Residual glucose (g/L)	Residual starch (g/L)	Total ABE produced (g/L broth)	Yield (g/g)	Productivity (g/L/h)
Glucose (control)	16.2	–	14.2	0.35	0.22
Food waste (81 g/L)	5.4	5.3	18.8	0.38	0.46
Food waste (129 g/L)	21.7	17.8	19.7	0.36	0.41
Food waste (181 g/L)	40.5	32.6	20.9	0.37	0.42
Food waste (228 g/L)	61.7	50.2	19.1	0.38	0.38
Food waste vacuum (129 g/L)	1.1	16.0	27.2	0.36	0.49

Fermentation of food waste medium at a higher concentration can reduce energy requirement, water consumption as well as volumes of process streams and processing equipment, thereby reducing butanol production cost [29, 30]. Next, ABE fermentations were performed with food waste medium at higher concentrations of 129, 181, and 228 g/L. All fermentations were rapid and completed within 48 h (Fig. 2b–d). At 48 h, total ABE in the medium was 19.7 and 20.9 g/L when the initial food waste concentrations were 129 and 181 g/L, respectively. These ABE concentrations were slightly higher than the ABE fermentation with food waste at a concentration of 81 g/L. When the initial food waste concentration increased to 228 g/L, total ABE in the broth decreased to 19.1 g/L (Fig. 2d). The decreased concentration in ABE was probably due to the high substrate inhibition to the culture, as previous studies reported that high substrate (glucose) would negatively affect ABE production [16]. During 48-h fermentation, the ABE productivities were 0.41, 0.42 and 0.38 g/L/h when the initial food waste concentrations were 129, 181, and 228 g/L, respectively. Fermentation of food waste at higher concentrations resulted in higher glucose during and after fermentation (Fig. 3). For the ABE fermentation with food waste at 129 g/L, the glucose concentration peaked (28.5 g/L) at 15 h, and stabilized at about 22 g/L after 24 h. For the ABE fermentation with food waste at 181 and 228 g/L, the glucose concentration increased consistently during the entire fermentation, implying that the glucose production rate by starch hydrolysis was higher than the glucose consumption rate by the culture. When the fermentations completed at 48 h, the residual glucose concentrations in the broth were 21.7, 40.5 and 61.7 g/L, respectively (Table 1). Furthermore, as the initial food waste concentrations increased from 129 to 228 g/L, the residual starch concentrations in broth after fermentation increased from 17.8 to 50.2 g/L, which again implies that *C. beijerinckii* was unable to completely hydrolyze the starch especially at high food waste concentrations. In these experiments, the ABE yields were 0.36, 0.37 and 0.38 g/g when the initial food waste concentrations were 129, 181, and 228 g/L, respectively (Table 1).

### Butanol fermentation in food waste medium with vacuum recovery

**Fig. 4:**
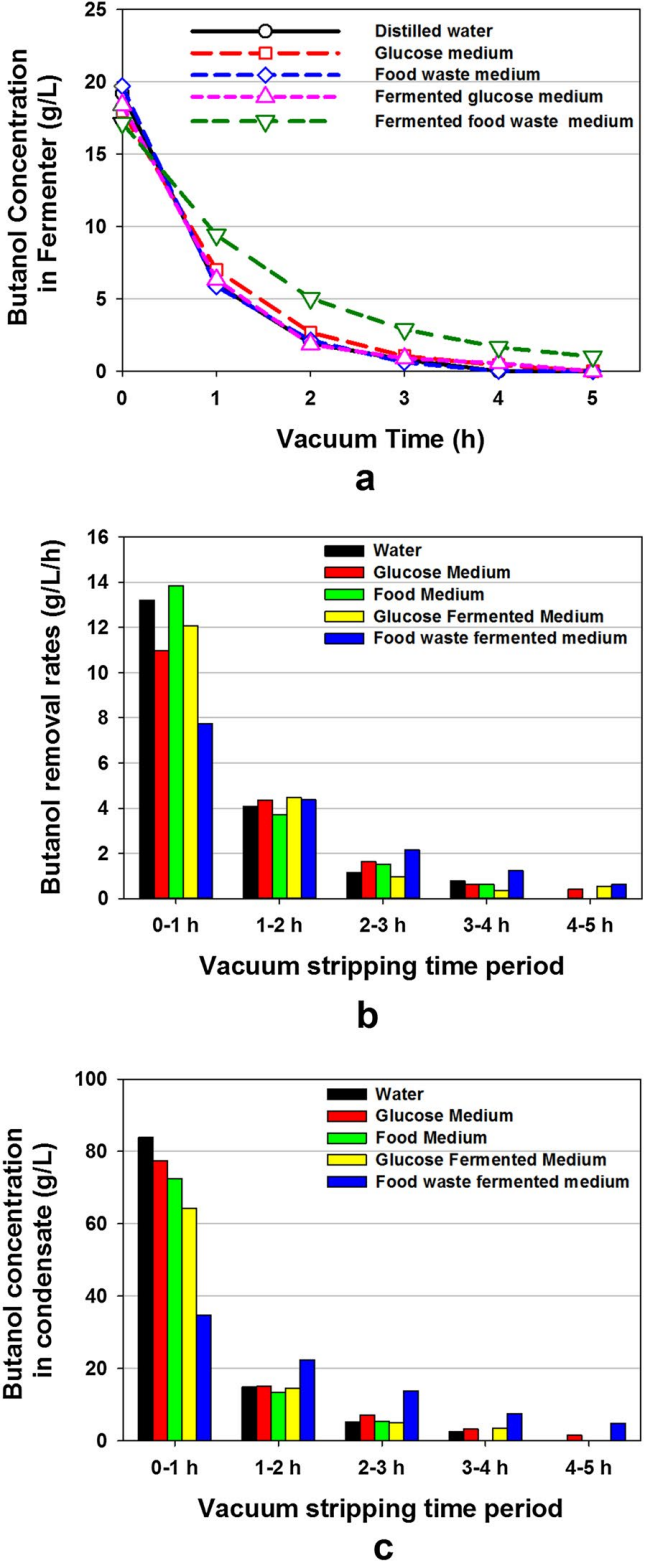
Butanol removal from different model solutions during 5-h vacuum stripping. **a** Butanol concentrations in different model solutions; **b** butanol removal rates from different model solutions; **c** butanol concentrations in condensates

To study the butanol removal characteristics with the designed vacuum stripping system, butanol removal experiments were conducted using model solutions to evaluate the effect of vacuum time and medium type on the removal efficiency. Figure 4a shows the decrease in butanol concentrations in the model solution with different types of media over 5 h vacuum stripping time, showing that the designed vacuum stripping system can effectively remove the butanol. The butanol concentration change profiles for the first four media were similar. The butanol concentrations decreased from 18 g/L to 6–7 g/L during the first 1-h vacuum stripping, with the butanol removal rates between 11 and 13 g/L/h. For the fermented food waste medium, the butanol removal rate at the first 1 h was lower (7.7 g/L/h) compared to the other four media (Fig. 4b). This could be due to the composition difference between fermented food waste medium and the other four media, since some compounds (acetic and butyric acids) in the media negatively impact butanol evaporation during the vacuum stripping [27]. Another possible reason may be presence of polysaccharides that are produced during fermentation, and they make fermented broth viscous. It was noticed that the fermented medium was more viscous than the other four media by observation. Removal of butanol from the viscous medium may be affected in two ways: (1) difficulty in controlling temperature at the set point which was 35 °C, and (2) slow diffusion of butanol from the bulk (inner layers) of the liquid to the surface due to increased viscosity caused by residual unhydrolyzed starch and polysaccharides produced by the culture. The first possibility was confirmed by the observation that the temperature of the fermented food waste medium was 33–34 °C during the vacuum stripping rather than the set point at 35 °C.

The butanol removal rates decreased rapidly with time during the 5-h vacuum stripping (Fig. 4b). For example, the butanol removal rates in the fermented food waste medium were 7.7, 4.4, 2.2, 1.2 and 0.6 g/L/h for the vacuum duration of 0–1, 1–2, 2–3, 3–4, and 4–5 h, respectively. The decreased removal rates were due to the decreased butanol concentrations in the model medium, as higher butanol concentrations result in higher butanol removal rates by the vacuum stripping [31]. The butanol concentrations in the condensate were much higher compared to the media (Fig. 4c), indicating that the designed vacuum stripping system had the capability to concentrate butanol in condensate. To make the process energy efficient, only 1 h of the vacuum stripping was applied to remove and recover butanol from the medium in the integrated simultaneous saccharification, fermentation and recovery process with food waste as substrate.

**Fig. 5:**
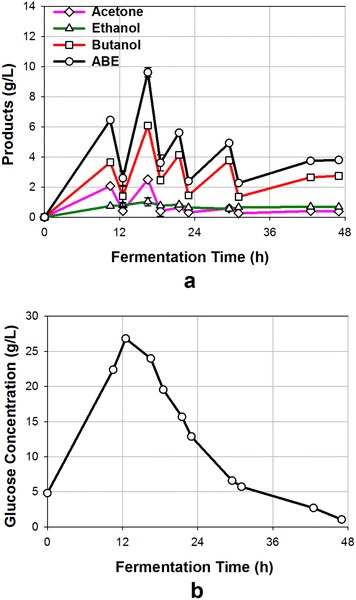
ABE fermentation of food waste at an initial concentration of 129 g/L in a batch fermenter coupled with simultaneous product recovery by vacuum stripping. **a** product concentrations in the broth at various fermentation times; **b** glucose concentrations in the broth at various fermentation times

ABE fermentation of food waste at high concentrations resulted in incomplete utilization of glucose, which was generally due to the accumulation of ABE at high concentrations that was toxic to the culture. To utilize all sugars in the medium, ABE should be removed from the broth simultaneously by the vacuum stripping during fermentation. An experiment was performed with food waste medium at an initial concentration of 129 g/L and fermentation was initiated. After a period of 11.5 h, as ABE concentration reached 6.5 g/L, product removal by vacuum stripping was started (Fig. 5a). After 1-h vacuum stripping, ABE concentration in the fermentation broth decreased to 2.6 g/L, thus showing the high ABE removal capability by this system (vacuum stripping). Vacuum stripping was also applied for 1 h at 17.5, 22 and 30 h during fermentation to remove ABE. Therefore, vacuum stripping was applied four times, with one hour at each time. Figure 5a shows that vacuum stripping successfully controlled the ABE concentrations in broth below 10 g/L, with butanol concentration below 6 g/L. The ABE removal rates by the vacuum stripping were between 2.7 and 6.0 g/L/h, depending on the ABE concentration in the fermentation broth. Gas stripping is another widely used technology to remove butanol from medium in ABE fermentation [21, 32–34]. This study showed that ABE removal rate by the vacuum stripping technology was much higher compared to the gas stripping technology, which typically has removal rates below 0.5 g/L/h [32, 35]. Due to the high ABE removal rates, vacuum pump was only required to run for a total of 4 h during the entire 47-h fermentation in this study; while gas stripping has to be applied for a much longer time to control the butanol concentration at a low level [16, 35]. Longer operating time required by the gas stripping technology leads to higher energy consumption for an associated blower and condenser, potentially incurring higher operating cost and resulting in higher butanol production cost [14]. Compared to gas stripping, the shorter operating time required by the vacuum stripping could be an effective alternative option to reduce the energy consumption for removing butanol from fermentation broth. Furthermore, vacuum stripping likely has lower capital investment compared to gas stripping technology [14].

**Fig. 6:**
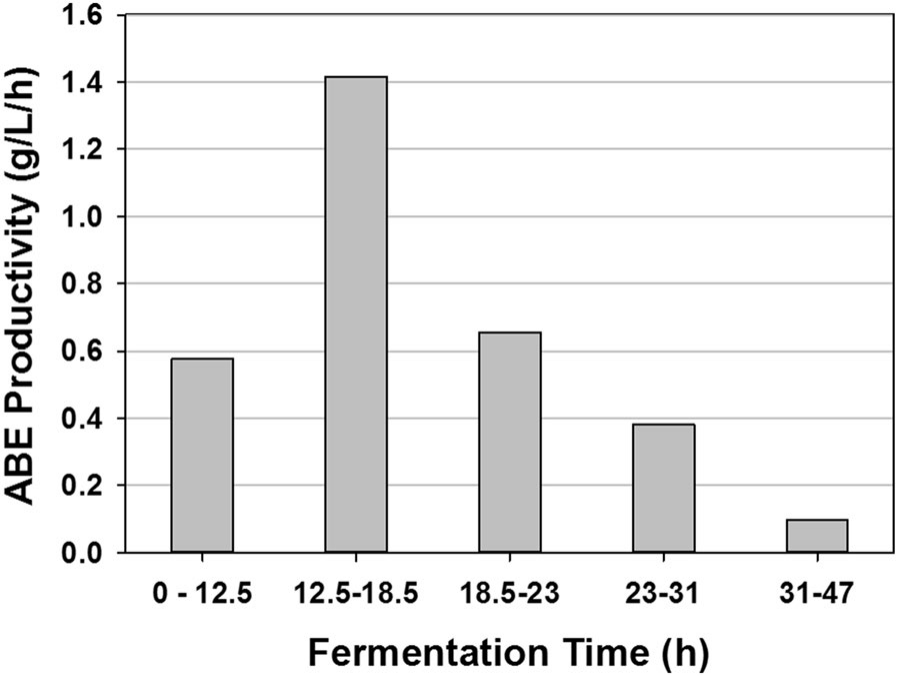
ABE productivity during different fermentation time periods in the integrated saccharification, fermentation and product recovery system

Fermentation stopped at 47 h as indicated by no increase in ABE concentration. At 47 h, the glucose concentration in the fermentation broth was 1.1 g/L, indicating near-complete utilization of the glucose (~98 %). The residual glucose concentration was as high as 21.7 g/L when the ABE fermentation was conducted at the same level of food waste concentration (129 g/L) without applying the vacuum stripping (Fig. 3). Therefore, vacuum stripping allows complete ABE fermentation at higher food waste concentrations by removing toxic products. The cessation of fermentation at 47 h was probably due to substrate limitation (near-to-zero glucose concentration) in the broth, considering that the fermentation rates (ABE productivity) were closely related to the glucose concentration (by comparing Fig. 5b with Fig. 6). At the end of fermentation, the residual starch in broth was measured at 16.0 g/L. ABE yield in this food waste vacuum fermentation was 0.36 g/g, which was comparable to the ones reported in Table 1.

**Fig. 7:**
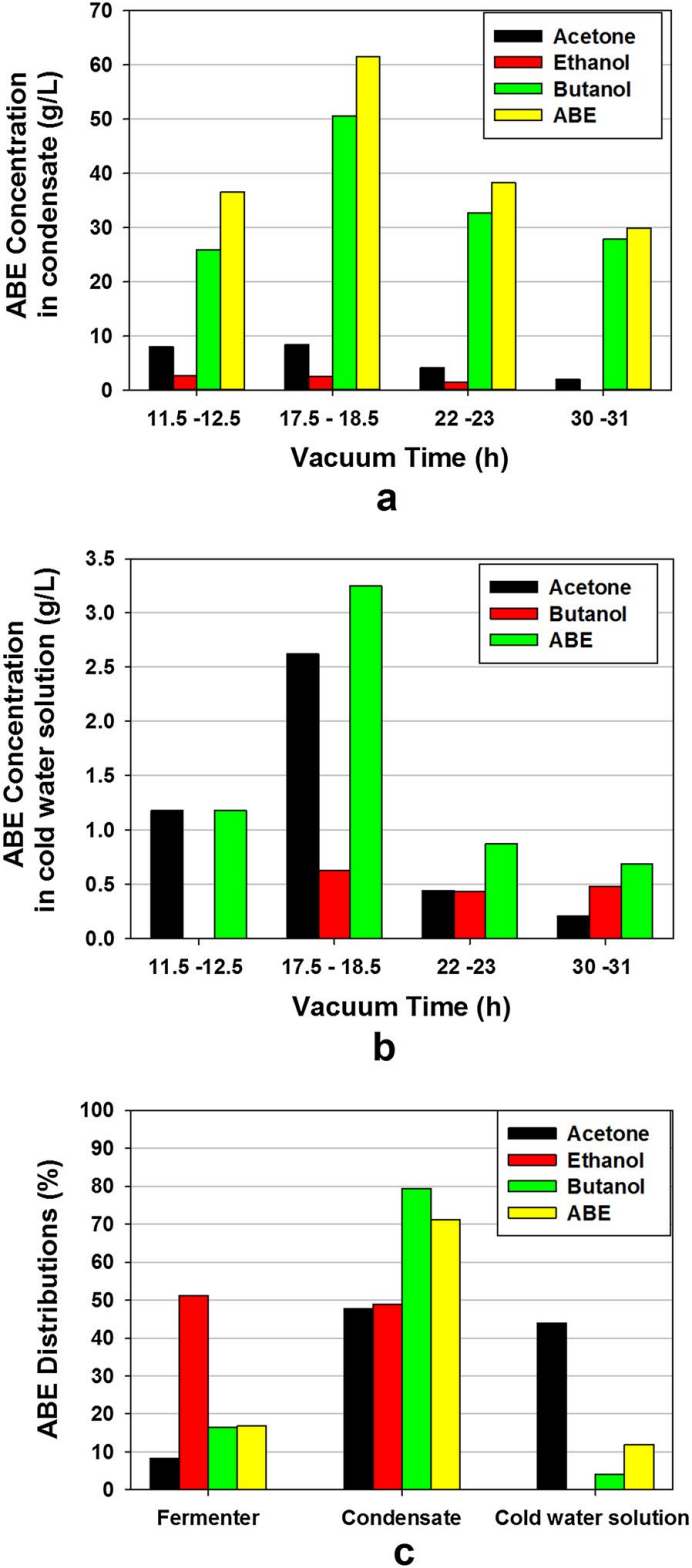
ABE concentrations and distributions. **a** ABE concentrations in the condensate; **b** ABE concentrations in cold-water solution; **c** ABE distributions in fermentation broth, condensate, and cold-water solution

In the experiment, both condensate and cold-water solution were collected and measured. The ABE concentrations in the condensates were between 29.9 and 61.5 g/L, with the butanol concentrations in the range of 25.9–50.6 g/L (Fig. 7a). These values were comparable to the 33 g/L ABE concentrations in the condensate in the previous vacuum fermentation study [27]. Selectivity is commonly used to evaluate pervaporation membrane performance for its capability to separate and concentrate the desired products from a mixture. However, it can also be used for evaluating the vacuum stripping [19]. For the present experiment, the ABE selectivities were 8.3, 9.8, 9.9 and 8.5, respectively, for the four vacuum stripping applications (11.5–12.5 h, 17.5–18.5 h, 22–23 h, and 30–31 h). It indicates that vacuum stripping not only removed the ABE from broth to reduce culture inhibition, but also concentrated the ABE in condensate. Increase in ABE concentration can have a substantial impact on energy saving. Previous study showed that, as the concentration of butanol increased from 12 to 19 g/L, the energy required for butanol distillation was decreased by 50 % [36].

During the experiment, some vaporized ABE was not condensed by the coiled condenser but was captured by bubbling the vapors/gas in the cold water. Figure 7b shows the ABE concentrations in the cold-water solution. The acetone concentration in the cold-water solution was exceptionally high compared to the butanol. The boiling point of acetone (56 °C) is lower than that of ethanol (78 °C), and butanol (118 °C), making it difficult to be condensed by the current condensing system. No ethanol was detected in the cold-water solution indicating that all evaporated ethanol was condensed.

By combining the ABE products in the fermentation broth, the condensate, and the cold-water solution, a total of 15.1 g ABE was produced, of which acetone, ethanol and butanol were 3.1, 0.9 and 11.1 g, respectively. The detailed distributions of ABE and each of the products are shown in Fig. 7c. In this integrated vacuum fermentation system, 17 % of the produced ABE remained in the fermentation broth, 71 % and 12 % were recovered in the condensate and the cold-water solution, respectively. For butanol, 80 % were recovered in the condensate, 17 % remained in the fermentation broth, and only 4 % captured in the cold-water solution. The condensation system was unable to effectively trap the acetone; therefore, a substantial fraction of acetone (44 %) was captured in the cold-water solution. This phenomenon was also reported by previous studies [27, 31], which used vacuum stripping to remove ABE from the glucose fermentation broth. Therefore, it is necessary to bubble the exit gas from the vacuum pump in the cold water to trap the uncondensed ABE, especially acetone. Operating the condensing system at a lower temperature (<1 °C) can condense more vaporized ABE, but the energy (electricity) cost to cool down the chilled liquid would be increased. This scenario should be evaluated by the detailed techno-economic analysis.

ABE productivity of the food waste vacuum fermentation ranged from 0.1 to 1.4 g/L/h, with an average value of 0.49 g/L/h (Fig. 6). This value was 109 % higher than that of the control fermentation (glucose based), and was about 20 % higher than that of the food waste (129 g/L) fermentation without applying vacuum stripping. The high productivity of vacuum fermentation was generally due to the combinations of superior substrate of food waste and reduced ABE inhibition to the culture by vacuum stripping. The reduced ABE productivity after 18.5 h was probably due to the low glucose concentration in the fermentation broth (Fig. 5b). The reader is informed that for the food waste fermentations, no hydrolytic enzymes were added as the culture was capable of hydrolyzing the starch contained in the food waste, which is an added advantage for butanol fermentation. The objectives stated in the introduction section have been successfully completed.

### Comparison to other studies

ABE fermentation of different feedstocks (wheat straw, corn stover, whey permeate, wood pulping, cassava bagasse, and glucose) with different strains to ABE has been investigated by different studies (Table 2). The final ABE concentrations without integrated product recovery system were between 9.4 and 22.7 g/L, mainly depending on different feedstocks and strains. In our study, the final concentrations were between 18.8 and 20.9 g/L, close to the highest ABE concentrations reported in other studies (Table 2). All studies (including this study) showed that the ABE yields were between 0.30 and 0.40 g/g, regardless of different strains used. The productivities in this study with food waste as substrate were between 0.38 and 0.46 g/L/h, higher than values published in most other studies. With the integrated product recovery system, the ABE yield remained more or less the same, but the fermentation productivities increased at different levels. The productivity in this study with vacuum stripping in batch fermentation increased to 0.49 g/L/h. This value was higher than the ones reported by other studies where batch fermentation processes were used (0.29 to 0.34 g/h/L), but lower than the ones where fed-batch fermentation processes were used (0.53–1.16 g/h/L). Therefore, the system in our study can be further improved by designing a fed-batch bioreactor, where concentrated food waste is fed to the reactor and toxic butanol is simultaneously removed by vacuum stripping during fermentation.

**Table 2:** Comparison of ABE fermentation with different typical feedstocks

Feedstock	Process mode	Culture	Fermentation without integrated product recovery			Fermentation with integrated product recovery			References
			ABE titer (g/L)	ABE yield (g/g)	Productivity (g/L/h)	ABE titer (g/L)	ABE yield (g/g)	Productivity (g/L/h)	
Wheat straw	Batch^a^	*C. beijerinckii* P260	9.4–13.1	0.37–0.42	0.14–0.27	<10^c^	0.41	0.31	[17]
Corn stover	Batch^b^	*C. beijerinckii* P260	14.2	0.30	0.22	~13.8	0.39	0.34	[19]
Switchgrass	Batch^a^	*C. saccharobutylicum* DSM 13864	22.7	0.40	0.63	–	–	–	[22]
Whey permeate	Batch^a^	*C. acetobutylicum* P262	11.3	0.39	0.22	~5.0	0.27	0.31	[37]
Wood pulping	Batch^a^	*C. beijerinckii* CC101	11.4	0.39	0.16	~11.0	0.44	0.25	[38]
Cassava bagasse	Fed-batch^a^	*C. acetobutylicum* JB200	15.4	0.34	0.39	~20.0	0.32–0.37	0.41–0.53	[21]
Glucose	Fed-batch^a^	*C. beijerinckii* BA101	17.6	0.39	0.29	~16.5	0.47	1.16	[39]
Food waste	Batch^b^	*C. beijerinckii* P260	18.8–20.9	0.36–0.38	0.38–0.46	~10.0	0.36	0.49	This study

^a^Fermentation integrated with gas stripping recovery system

^b^Fermentation integrated with vacuum stripping recovery system

^c^The highest ABE concentration in fermentation broth during fermentation with integrated product recovery process

## Conclusions

In these studies (for the first time) it was demonstrated that food waste is a superior feedstock for producing butanol using *C. beijerinckii*. Compared to costly glucose, ABE fermentation of food waste has several advantages including lower feedstock cost, higher productivity, and less residual sugars. The final ABE concentration in food waste fermentation was 18.9 g/L, while the final ABE concentration in glucose fermentation was only 14.2 g/L. The ABE productivity of the food waste fermentation was 0.46 g/L/h, which was over 100 % higher than the glucose fermentation. Additionally, food waste fermentation to butanol did not require supplementation of hydrolytic enzymes, which is considered to be an economical advantage. The hydrolytic enzymes were secreted by the culture.

ABE fermentation integrated with novel vacuum stripping technology successfully controlled the butanol concentration in broth below 6 g/L, and allowed near-complete utilization of glucose when the food waste concentration was as high as 129 g/L. Fermentation of food waste at higher concentrations with the vacuum stripping technology can potentially reduce energy requirement, water consumption, and volumes of process streams and processing equipment, thereby reducing butanol production cost. Efficient utilization of food waste for butanol production provides a promising approach to solve the energy and environmental sustainability issues.

## Methods

### Food waste

The food waste was obtained from a local retail store in Urbana, Illinois, USA, and mainly contained mashed potatoes, sweet corn and white bread, and was used as a model food waste. The composition of the collected food wastes were analyzed and shown in Table 3. The procedures of the composition measurement are provided in Analyses section. The high starch content in the food waste sample was very similar to the sample reported in the previous study [40]. The received food waste was pulverized and mixed using a fruit/vegetable mixer for 3 min, analyzed for moisture content [41], and stored at −20 °C for the following experiments. Frozen food waste samples were transferred from the freezer and placed at room temperature for 12–14 h before experiments to allow it to thaw.

**Table 3:** Food waste composition

	Starch (d.b.)^*a*^	Glucose (d.b.)	Protein (d.b.)	Oil (d.b.)	NDF (d.b.)
Percentage (%)	63.5 ± 1.8	4.3 ± 0.6	13.9 ± 0.1	4.1 ± 0.2	5.2 ± 0.9

*NDF* neutral detergent fiber

^a^ *d.b* Dry basis. The starch, glucose, protein, oil and NDF concentrations are based on dry matter

### Culture and cell propagation

*Clostridium beijerinckii* P260 was a generous gift from Professor Davis Jones (University of Otago, Dunedin, New Zealand). Spores of the culture were stored in distilled water in a refrigerator at 4 °C. For *C. beijerinckii* spore activation, 100 µL of spores were heat shocked at 75 °C for 2 min, and 20 µL of the heat-shocked spores were transferred to cooked meat medium (CMM; Difco™; Becton, Dickinson, and Company, Sparks, MD, USA). To prepare liquid CMM, 3.5 g of CMM pellets, and 0.6 g of glucose (Sigma Chemicals, St. Louis, MO, USA) were suspended in 35 mL distilled water in a 50-mL screw-capped Pyrex™ bottle. The mixture was autoclaved at 121 °C for 15 min followed by cooling to 30 °C. After spore inoculation, the bottles were placed in a 3-L anaerobic jar (BBL GasPak™, Sparks, MD, USA). Anaerobic conditions inside the jar were developed using BD GasPak™ EZ (Sigma Chemicals, USA) envelopes with indicators. Prior to placing the bottles in the anaerobic jar, caps were loosened to help exchange of gases between the jar and the medium in the bottles. Then the jar was placed in an incubator at 35 °C for 16–18 h and the culture was used as the first-stage inoculum. Following that, 7 mL of the first-stage culture was transferred to 100 mL of the second-stage medium (P2 medium). P2 medium was prepared by adding 3 g of glucose, 0.2 g of yeast extract (Bacto-Dickinson & Co., Sparks, MD, USA) to 100 mL of distilled water in a 125-mL screw-capped bottle, followed by autoclaving at 121 °C for 15 min. After autoclaving, 1 mL each of filter-sterilized stock solutions (mineral, buffer and vitamin) were added to P2 medium. Cell growth in P2 medium was allowed at 35 °C for 6–8 h under anaerobic condition. Followed by culture preparation in P2 medium, actively growing culture was transferred to the fermentation medium. The fermentation medium preparations are described below.

### Production of butanol in conventional fermentation

The conventional batch fermentation studies were conducted in 1-L Pyrex™ screw-capped bottles containing about 600 mL medium. Fermentation with glucose at a concentration of 56.7 g/L was conducted as a control experiment. For glucose medium preparation, 56.7 g/L of glucose and 1 g/L of yeast extract were sterilized at 121 °C for 15 min followed by cooling to room temperature. After cooling, 6 mL of each of stock solutions (vitamin, buffer, and mineral) were added to the medium. Then the medium was inoculated with 40 mL of actively growing second-stage culture developed in P2 medium. The bottles were then placed in an anaerobic chamber (Coy Lab Products Inc., Grass Lake, MI, USA) to start fermentation at 35 °C. During fermentation, 1.5 mL samples were taken for sugar and ABE measurement. The details of measurements are described in Analyses section. Fermentation was conducted until culture ceased ABE production, which was indicated by no increase in ABE concentrations. Each batch fermentation was conducted in duplicate.

Studies with food waste as substrate at various levels (81 to 228 g food waste/L) were conducted in a 1-L Pyrex™ screw-capped bottle containing about 600 mL medium. Various concentrations of food waste medium and 1 g/L yeast extract were sterilized at 121 °C for 15 min followed by cooling to room temperature. Then 6 mL of each stock solution (vitamin, buffer, and mineral) was added. The food waste concentrations in the prepared samples were 81, 129, 181, and 228 g/L. In the food waste medium at a concentration of 81 g/L, the equivalent glucose concentration (existing glucose plus hydrolyzed glucose by assuming complete hydrolysis of starch in food waste) was 60.1 g/L, which is comparable to the 56.7 g/L of glucose concentration in the control experiment. Then the medium was inoculated with 40 mL of stage-two culture developed in P2 medium, followed by fermentation at 35 °C in the anaerobic chamber. During fermentation, 1.5 mL samples were taken for sugar and ABE measurement until fermentation ceased. Each experiment was conducted in duplicate.

### Production of butanol by integrated vacuum fermentation

**Fig. 8:**
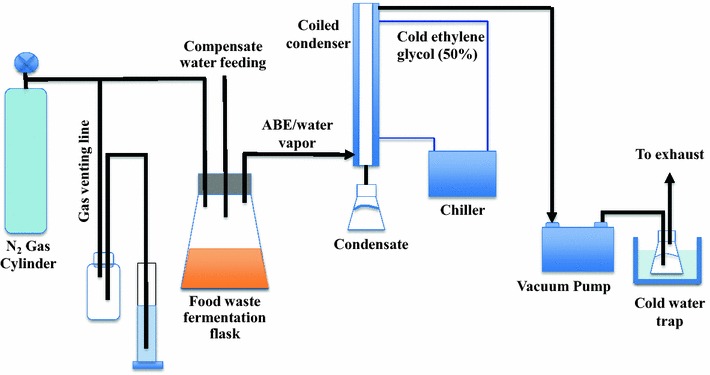
Schematics of in situ ABE recovery from fermentation broth by vacuum stripping. *Arrows* show direction of ABE flow. The food waste vacuum fermentation system consisted a 2-L fermentation flask, a vacuum pump, a condensation system with a chiller and a coiled condenser and a cold-water trap. During the vacuum application, ABE and water vapors evaporated from the fermentation broth and condensed in the condensation system. The escaped (uncondensed) ABE and water vapors were captured in the cold-water trap

The integrated vacuum fermentation system is illustrated in Fig. 8. The integrated vacuum fermentation was performed in a 2-L glass flask that had ports for vacuum, gas inlet and outlet, and distilled water addition. The bottle and medium temperature was controlled by putting it in a heated water bath at 35 °C. To allow vacuum stripping, the flask was connected to a vacuum pump. When vacuum stripping was applied, fermentation broth in the flask boiled at the fermentation temperature generating ABE and water vapors. The evaporated ABE and water vapors were condensed by passing them through a coiled condenser, which was cooled with chilled liquid (50 % v/v ethylene glycol in distilled water) to 1 °C using a refrigerated circulating bath (Thermo Haake C-35P, Cole Parmer, Vernon Hills, IL, USA). The condensate in the 250-mL conical flask was pumped out into a solvent collector using a peristaltic pump. Vacuum was generated with oilless vacuum pump at 4.7 kPa (28.5 in Hg gauge pressure) (Model DAA, Gast Manufacturing Inc., Benton Harbor, MI, USA). Vacuum pump exhaust (flue gas) was bubbled in a 300-mL cold-water solution chilled with iced water, to collect escaping (uncondensed) ABE vapors. After each vacuum stripping, pressure inside the fermentation bottle was restored to atmospheric pressure by injecting oxygen-free N_2_ gas, and the excess N_2_ gas vented through the gas venting line shown in Fig. 8. To evaluate and characterize the designed vacuum stripping system for removing butanol from fermentation medium, the vacuum removal of butanol was conducted at 35 °C using model solutions. Five different model solutions were used to characterize the effects of medium components on butanol removal by vacuum. These model solutions were (1) butanol in 1 L distilled water; (2) butanol in 1 L 60 g/L glucose solution; (3) butanol in 1 L 81 g/L food waste medium; (4) butanol in 1 L fermented glucose medium (glucose concentration of 60 g/L before fermentation); and (5) butanol in 1 L ABE fermented food waste (food waste concentration of 81 g/L before fermentation). The butanol concentration in each model solution was adjusted to approximately 18 g/L. Vacuum stripping was applied to the model solution for 5 h during which samples were taken from the fermenter every hour for analysis. After each sampling, an appropriate amount of distilled water (100–125 mL) was added to compensate for the evaporated butanol/water by the vacuum stripping; therefore, the volume of the model solution was maintained at 1 L.

The vacuum fermentation of food waste to produce ABE was performed in the system described above. The conventional batch fermentation results showed that more than 60 g/L glucose cannot be utilized by *C. beijerinckii* due to butanol inhibition (Fig. 3). Therefore, the medium with the food waste concentration of 129 g/L was conducted by vacuum fermentation where butanol was removed from the broth simultaneously. The medium preparation was as described above for the conventional food waste fermentation. After autoclaving and cooling, the food waste medium was inoculated with 40 mL stage-two culture developed in P2 medium and fermentation was initiated. Anaerobic conditions inside the flask were maintained by sweeping oxygen-free N_2_ gas across the medium surface until vacuum was applied. The fermentation was allowed to proceed for 11.5 h during which butanol concentration reached 3.6 g/L, and was followed by ABE recovery by vacuum stripping. The generated ABE and water vapors were cooled in the coiled condenser. Vacuum stripping was applied for 1 h at each of 11.5, 17.5, 22, and 30 h during fermentation. The aim of the vacuum stripping was to keep the butanol concentration in fermentation broth at low levels to relieve butanol toxicity to *C. beijerinckii*, thus allowing the culture to use all sugars and produce more ABE. Samples were taken before and after each vacuum stripping to monitor the immediate effect of vacuum stripping on the ABE concentrations in fermentation broth.

### Analyses

The glucose and starch concentrations in the food waste were measured by the modified dilute acid method [42]. The crude protein, oil, and ash concentrations in the food waste were determined according the AOCS Official and Tentative Methods Ba 4e-93, Am 5-04 and Ba 5a-49, respectively. The neutral detergent fiber concentration in the food waste was determined using the ANKOM200/220 Fiber Analyzer (ANKOM Technology, Macedon, NY, USA) [43]. Fermentation products (acetone, butanol, and ethanol) were analyzed by gas chromatography (GC) (6890 N; Agilent Technologies, Wilmington, DE, USA) using a packed column as described previously [35, 44]. The samples were centrifuged at 13,000 rpm for 5 min and the supernatants were diluted fourfold with distilled water before injecting into the GC. The GC was equipped with an auto-sampler and an integrator. Sugars were measured using high-performance liquid chromatography (HPLC) equipped with an automatic sampler/injector (Waters Corporation. Milford, MA, USA). The ion exchange columns (BioRad Aminex HPX-87H, and HPX-87P) were used. For sugar analysis, the samples were centrifuged at 13,000 rpm for 10 min and the supernatants were filtered through 0.2-µm syringe filters (Waters Corporation. Milford, MA, USA) [45]. After fermentation, the broth was analyzed for residual starch concentration by the modified dilute acid method [42]. ABE productivity was calculated as total ABE (present in the fermentation broth plus condensate) produced in g/L divided by the fermentation time and is expressed as g/L/h. ABE yield was calculated as total ABE produced divided by total sugar/starch utilized. Butanol/ABE selectivity (a measure of preferential removal of butanol/ABE over other components in the mixture) was calculated as:

1 $$\alpha = \frac{y/(1 - y)}{x/(1 - x)}$$

where *x* and *y* are weight fractions of butanol/ABE in fermentation broth and condensate, respectively.

## Abbreviations

GC: gas chromatography
SSFR: simultaneous saccharification fermentation and recovery
HPLC: high-performance liquid chromatography
